# Mediators of increased blood flow in porcine skin

**DOI:** 10.1155/S0962935192000115

**Published:** 1992

**Authors:** H. D. Moore, F. M. Cunningham

**Affiliations:** 1 Department of Veterinary Basic Sciences The Royal Veterinary College Hawkshead Lane North Mymms, Hertfordshire AL9 7TA UK

## Abstract

Nicotinates and benzalkonium chloride (B.Cl) cause inflammatory changes in human skin, thought to be dependent upon prostaglandin formation. This study has examined the effects of hexyl-nicotinate (HN) and B.Cl on blood flow in porcine skin. The role of prostaglandins and interleukin (IL)-1 in the blood flow response has been investigated. Blood flow was increased by both HN and B.Cl, the response to B.Cl being more protracted. Cyclooxygenase inhibitor pretreatment reduced these responses. IL-1-like biological activity was identified in normal porcine epidermis and the amounts recovered from inflamed skin were similar. Thus prostaglandin formation in HN or B.Cl-induced inflammation, if IL-1 dependent, is not associated with the loss of significant amounts of the cytokine from the epidermis.


Mediators of Inflammation 1, 55–59 (1992)

### Mediators of increased blood flow in porcine skin

H. D. Moore and F. M. Cunningham[CA]

Department of Veterinary Basic Sciences,
The Royal Veterinary College, Hawkshead Lane,
North Mymms, Hertfordshire, AL9 7TA, UK

[CA] Corresponding Author

**Nicotinates and benzalkonium chloride (B.Cl) cause inflammatory changes in human skin, thought to be dependent upon prostaglandin formation. This study has examined the effects of hexyl-nicotinate (HN) and B.Cl on blood flow in porcine skin. The role of prostaglandins and interleukin (IL)-1 in the blood flow response has been investigated. Blood flow was increased by both HN and B.Cl, the response to B.Cl being more protracted. Cyclooxygenase inhibitor pretreatment reduced these responses. IL-1-like biological activity was identified in normal porcine epidermis and the amounts recovered from inflamed skin were similar. Thus prostaglandin formation in HN or B.Cl-induced inflammation, if IL-1 dependent, is not associated with the loss of significant amounts of the cytokine from the epidermis.**

**Key words:** Cutaneous blood flow, Cutaneous inflammatory response, Interleukin-1, Porcine skin, Prostaglandins

### Introduction

Nicotinate esters have, for many years, been included in rubefacient preparations, which are thought to relieve pain by acting as counter irritants. Topical application of nicotinates to human skin has been shown to cause erythema, an increase in blood flow, oedema and leucocyte infiltration.[1,2] These compounds, together with benzalkonium chloride (B.Cl), a skin disinfectant which has also been shown to produce inflammatory effects in human skin,[3] have been used to study the mechanisms underlying aspects of the cutaneous response to local irritation. Thus prostaglandins (PGs) have been implicated in the pathogenesis of the inflammatory response to nicotinates and B.Cl. Increased amounts of $PGE_2$ have been recovered from suction blisters raised on Trafuril® (tetrahydrofurfuryl-nicotinate) and B.Cl-inflamed skin and inhibitors of PG synthesis shown to reduce both the erythematous and cellular responses to the nicotinate.[2–4] Whether PG formation occurs as a direct result of damage to cells in the epidermis or dermis by such compounds, or indirectly via the release of a second mediator(s), has not been determined.

Normal human skin contains large amounts of the cytokine interleukin (IL)-1 and injurious stimuli have been reported to cause the synthesis or release of membrane bound IL-1 from keratinocytes *in vitro*.[5–10] Since IL-1 can induce the formation of vasodilator PGs in cultured keratinocytes and endothelial cells,[11,12] this cytokine may play a role in the PG production that accompanies the inflammatory response to nicotinate esters and B.Cl.

The structure of porcine skin closely resembles that of man.[13] Studies of such physiological and pathophysiological processes as epidermal cell kinetics, ultraviolet light induced skin damage and responses to inflammatory mediators have also yielded similar results to those obtained in human skin.[14–16] We now report a method which has been devised to examine the effects of topically applied hexyl-nicotinate (HN) and B.Cl on blood flow in mini-pig skin. The role of PGs and IL-1 in mediating such responses has also been investigated.

### Materials and Methods

*Reagents:* IL-1α was a gift from Dr. S. Gillis, Immunex Corporation, USA, and flunixin meglumine (Finadyne paste) from Dr. P. Smitherman, Schering-Plough, UK. Tritiated $PGE_2$ (specific activity 168.7 Ci $mmol^{-1}$) was purchased from New England Nuclear, UK and $^3H$-thymidine (specific activity 25 Ci $mmol^{-1}$) from Amersham International, UK. $PGE_2$ antibody was supplied by ICN Biomedicals, UK. All other reagents were purchased from Sigma Chemical Co., UK or Aldrich Ltd., UK and were of Analar grade.

*Measurement of cutaneous blood flow:* Mini-pigs (Gottingen strain; 15–25 kg) were trained to sit in a specially constructed box with a moveable side, comprised of removable bars, to enable blood flow to be measured with a laser Doppler flowmeter (Pf2b, Perimed, Sweden). Circular sites of 3.5 cm diameter were marked on the flanks, which had been clipped the previous day, and background blood flow readings obtained. Fifty μl of vehicle (dimethylsulphoxide (DMSO) and propylene

© 1992 Rapid Communications of Oxford Ltd glycol:isopropanol (PG:IPA;60:40) for B.Cl and HN respectively) was then applied topically under occlusion for 30 min. HN, B.Cl or vehicle (50 $\mu$l) were next applied under occlusion for a further 15 min and blood flow recorded at regular intervals until the responses reached a peak. Five readings were obtained at each site and the mean value calculated for each time point. Ten per cent solutions of HN and B.Cl were used as preliminary experiments had shown that 5 mg/site was the smallest amount of either compound that produced consistent increases in blood flow in all the pigs tested.

*Effect of cyclo-oxygenase inhibition on HN and B.Cl-induced inflammatory responses:* Inter-site variation in the blood flow response was first examined by applying HN to two sites on each of four pigs. HN and PG:IPA were next applied to two different sites and blood flow responses measured prior to oral administration of 5 mg kg$^{-1}$ flunixin meglumine (Finadyne paste). HN and PG:IPA were then applied to two further, matched, sites on the opposite flank, 2.5–4.5 h after drug administration. A 5 mg kg$^{-1}$ dose of flunixin was chosen because, when administered to calves at a dose of 4.4 mg kg$^{-1}$, the drug inhibited cyclo-oxygenase product formation in inflammatory exudates by more than 98% for at least 12 h (P. Lees, Royal Veterinary College; personal communication). In a second, similar, experiment in the same four pigs, blood flow responses to B.Cl and DMSO were examined before and after flunixin administration although, in view of the protracted blood flow response to B.Cl, pre- and post-drug measurements had to be carried out on successive days. The increases in blood flow caused by HN and B.Cl were then compared to pre-treatment values. Skin samples were taken from two pigs for measurement of $PGE_2$, 4 and 7 h after flunixin administration.

*Skin sampling for measurement of $PGE_2$ and IL-1-like activity:* Samples were first obtained to determine background levels of $PGE_2$ and IL-1-like biological activity in normal porcine skin. Inter-site variation in the recovery of these mediators was also measured. Local anaesthetic (1% lignocaine) was injected and skin samples collected 10 min later using a motor-driven keratotome (Storz, USA). The keratotome was set to a depth of 0.3 mm (epidermal and dermal tissue) when collecting samples for estimation of $PGE_2$ and 0.1 mm for measurement of epidermal IL-1. The samples were snap-frozen in liquid nitrogen and stored at $-20$°C until required for analysis.

In two separate experiments HN, B.Cl and the appropriate vehicles were applied to sites on the flanks of four mini-pigs. Samples were obtained from treated sites at the time of the maximum increase in blood flow caused by HN or B.Cl and the amount of IL-1-like activity in inflamed skin compared to that in vehicle treated sites. PGs could not be measured in inflamed skin since both HN and B.Cl were found to prevent PG extraction from the skin.

*Preparation of samples for measurement of $PGE_2$:* Frozen skin samples were powdered in liquid nitrogen and 50 mg aliquots added to 1 ml absolute ethanol. The samples were then homogenized three times, the supernatants pooled and the ethanol evaporated under a stream of nitrogen. After reconstituting in 10 ml Tris buffer (0.01 M, pH 7.0) and acidifying to pH 2–3 with 1 M citrate, samples were passed through prepared non-polar columns (functional group ethyl, 100 mg capacity, Bond-Elut) which retain $PGE_2$. The eluates were discarded, the columns washed with 4 × 250 $\mu$l toluene:dichloromethane (60:40) and eluted with 4 × 250 $\mu$l ethyl acetate:acetic acid (98:2). After evaporation of the solvents under nitrogen the samples were reconstituted in buffer for measurement of $PGE_2$ by radioimmunoassay (RIA).

*Preparation of samples for measurement of IL-1-like activity:* Aliquots of powdered skin were homogenized three times in RPMI-1640 buffer (15 mg in 0.8 ml). The supernatants from successive homogenizations were pooled, passed through a 0.2 $\mu$m filter and serial dilutions prepared. Epidermal IL-1-like activity was quantitated by use of a two-stage bioassay requiring EL4-NOB-1 and CTLL cells, which has been described in detail elsewhere.[17] The IL-1-like activity in porcine epidermal homogenates was quantitated by reference to a standard curve to human recombinant IL-1$\alpha$ carried out in the same assay and results expressed as pg human recombinant IL-1$\alpha$ equivalents/mg wet weight skin. Porcine IL-1$\alpha$ has been reported to be equivalent in activity to murine IL-1$\alpha$ in this assay system although porcine IL-1$\beta$ is two orders of magnitude less active.[18] Human recombinant IL-1$\alpha$ and $\beta$ (Immunex) are equivalent in activity to murine IL-1$\alpha$ (A.J.H. Gearing, personal communication).

*Anion-exchange chromatography:* Aliquots (37.5 mg) of powdered porcine epidermis were homogenized in 3 × 2 ml 20 mM Tris acetate buffer, pH 4. The supernatants were pooled and approximately one third of the sample then purified on a 7.5 cm × 7.5 mm TSK-DEAE-5-PW column (BioRad, UK), eluted with a gradient of 20 mM Tris acetate buffer over 20 min at a flow rate of 1 ml min$^{-1}$, as previously described for psoriatic scale and normal heel callus.[19] One ml fractions were collected, evaporated overnight and stored at $-20$°C until required for analysis in the IL-1 bioassay. Each fraction was reconstituted in 0.3 ml RPMI-1640 buffer and assayed at a dilution of 1:10.

### Results

*Effect of HN and B.Cl on blood flow:* Topical application of both HN and B.Cl caused a significant increase in blood flow, when compared to the vehicle and the response was usually accompanied by visible erythema. The time taken to reach the maximum response was consistent in all animals (Table 1), however, as illustrated in Fig. 1, the time course of the response to the two compounds differed. The B.Cl-induced increase in blood flow was not usually seen until at least 2 h, peaked at 4–6 h and often remained above background for more than 7 h. In contrast HN caused an increase in blood flow which was evident as early as 20 min after application, became maximal at 40–60 min and was approaching background values by 90 min. DMSO and PG:IPA caused a small increase in blood flow in some animals which persisted for no more than 30 min.

In addition to causing an increase in blood flow B.Cl also provoked oedema formation in nine of the twelve pigs. Small blisters were also seen in some pigs approximately 10 h after application of B.Cl.

*Effect of flunixin on HN and B.Cl-induced increases in blood flow:* Administration of flunixin inhibited the maximum blood flow response to HN by 46.4 ± 1.3%, a statistically significant reduction (Table 2). In addition the erythema that accompanied the increase in blood flow was abolished by drug treatment. Blood flow responses to HN at different sites in individual animals did not vary by more than 22%.

The blood flow response to B.Cl was also inhibited by flunixin treatment but the reduction did not achieve statistical significance (Table 2). This is likely to be due to the variability of the responses obtained since, although in two of the four pigs the increase in blood flow was completely

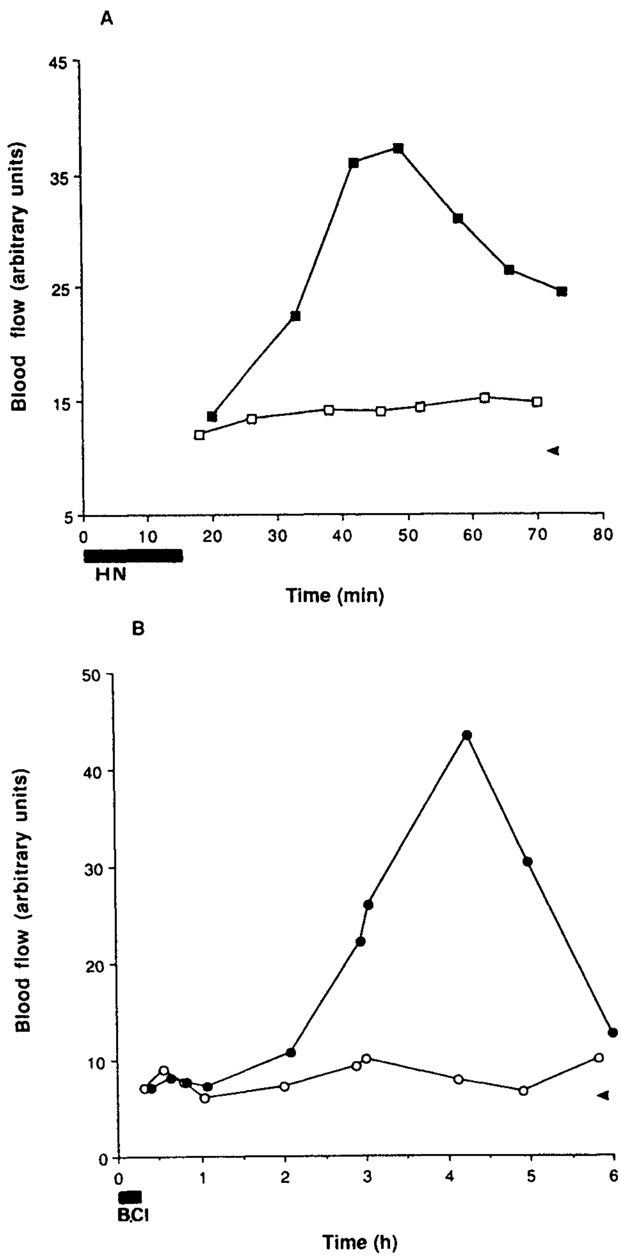

FIG. 1. Blood flow after topical application of A: HN (■—■) and PG:IPA (□—□) and B: B.Cl (●—●) and DMSO (○—○) to the skin of one pig. (◄) denotes background blood flow. The solid bar indicates the period of application of HN or B.Cl under occlusion (15 min).

**Table 1.** Effect of HN and B.Cl on blood flow in mini-pig skin

| | *n* | Time to reach maximum blood flow (min) | Maximal increase in blood flow |
|---|---|---|---|
| HN | 12 | 50 ± 3.3 | *43 ± 7 |
| PG:IPA | 12 | | 15.0 ± 3 |
| B.Cl | 12 | 246 ± 24 | *46 ± 6 |
| DMSO | 12 | | 15 ± 3 |

Results are expressed as mean ± SEM blood flow (arbitrary units) * = $p < 0.001$ versus vehicle (paired Student's *t*-test).

**Table 2.** Effect of flunixin on HN and B.Cl-induced blood flow responses

| | | [a]Maximal relative blood flow | |
|---|---|---|---|
| | *n* | Pre-flunixin | Post-flunixin |
| HN | 4 | 2.5 ± 0.1 | *1.3 ± 0.1 |
| B.Cl | 3 | 4.2 ± 0.7 | 1.6 ± 0.2 |

$^a$ Relative blood flow $= \frac{\text{blood flow + HN or B.Cl}}{\text{blood flow + vehicle}}$

* = $p < 0.05$ versus pre-treatment values (paired Student's *t*-test)

inhibited, as were visible signs of erythema and oedema, in the third there was only a 35% reduction in the response. In addition the fourth pig did not respond to 10% B.Cl and application of a 15% solution caused early oedema formation which precluded measurement of blood flow. Flunixin had no effect on the development of oedema in this pig.

Keratotome biopsies of normal porcine skin obtained from six pigs contained 1.18 ± 0.09 ng $PGE_2$/mg wet weight tissue (mean ± SEM). These values have not been corrected for recovery which was found to be 86 ± 2.6% (mean ± SEM; $n = 26$ columns). Inter-site variation in $PGE_2$, determined by measuring the amounts present in three samples from each of four pigs, was 12.2 ± 5.0% (mean ± SEM). $PGE_2$ levels in flunixin treated animals, measured after 4 and 7 h, were below the detection limit of the RIA (<15 pg/mg wet weight of tissue).

*IL-1-like activity in porcine epidermis:* IL-1-like activity was detected in 0.1 mm depth keratotome biopsies obtained from mini-pig skin (408 ± 80 pg IL-1α equivalents/mg wet weight tissue; mean ± SEM; $n = 8$). Stratum corneum also contained IL-1-like biological activity (459 ± 144 pg IL-1α equivalents/mg wet weight tissue; mean ± SEM; $n = 4$). Inter-site variation was determined by measuring the IL-1-like activity in three samples from each of four pigs and found to be 15.2 ± 2.9%. Assay of one sample from each of three pigs on four separate occasions showed the inter-assay variation to be 8.1 ± 2.3% (mean ± SEM).

The amount of IL-1-like activity in HN and B.Cl treated sites at the time of the maximum increase in blood flow was found to be similar to that in vehicle treated skin (Table 3). Neither HN nor B.Cl

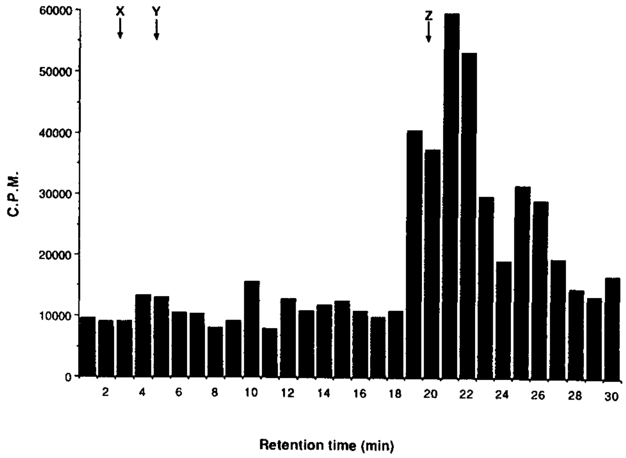

FIG. 2. IL-1-like biological activity obtained on anion exchange chromatographic separation of porcine epidermis. The retention times of pI markers are shown (X, myoglobin, pI 6.76, 7.16; Y, carbonic anhydrase, pI 5.85; Z, β-lactoglobulin, pI 5.13). One minute fractions were collected, evaporated overnight and reconstituted in 0.3 ml RPMI-1640 prior to bioassay. Samples were assayed in triplicate following 1:10 dilution.

**Table 3.** Effect of HN and B.Cl on IL-1-like activity in mini-pig skin

| Treatment | $n$ | IL-1 (pg human IL-1α equivalents/mg wet weight tissue) |
|---|---|---|
| PG:IPA | 4 | 579 ± 139 |
| HN | 4 | 413 ± 31 |
| DMSO | 4 | 359 ± 104 |
| B.Cl | 4 | 361 ± 89 |

Results are expressed as mean ± SEM.

interfered with the bioassay at the dilutions used to assay the samples (typically 1:64 to 1:2048; data not shown).

*Anion exchange chromatography:* Figure 2 shows a representative trace obtained after purification of porcine epidermal IL-1-like activity by anion exchange chromatography. Biological activity in the EL4-NOB-1 assay was only obtained in fractions eluting in the acidic region. Similar results were obtained in a further two experiments (data not shown). In view of the broad nature of the peak of IL-1-like activity and the steep transition from neutral to acid pH obtained with the Tris acetate gradient system used, a pI value cannot be assigned with any accuracy. Nevertheless it seems likely that the biological activity in the pI4–5 region is due to porcine IL-1α (pI5).

### Discussion

A method has been developed for quantitative measurement of the effects of topically applied chemical irritants and nicotinate esters on blood flow in mini-pig skin. Both of the compounds used in this study, HN and B.Cl, significantly increased blood flow. The time course of the responses, however, differed, one explanation for this being a difference in the percutaneous absorption characteristics of HN and B.Cl. The maximal increase in blood flow produced by HN was seen within an hour whereas B.Cl caused a much more protracted increase in blood flow that did not reach a peak until about 4 h after administration. Similar time courses for erythematous and blood flow responses have been reported in man, although HN was applied to human skin in lotion and B.Cl as an aqueous solution added to lyofoam within a skin chamber, rather than the solvents which were used when applying these compounds to porcine skin. Despite this, in man the increase in blood flow caused by HN was reported to be maximal after some 20 min and to return to baseline by 80 min.[1] The erythema seen after application of B.Cl has been described as being most intense after 4 h.[3]

The reduction in the blood flow response to HN and B.Cl observed in porcine skin after pretreatment with the cyclo-oxygenase inhibitor flunixin meglumine suggests that, as in man,[2-4] PGs are involved in mediating such responses. However, the mechanism by which such PG formation is brought about has yet to be established. HN or B.Cl could, for example, stimulate PG production directly or, alternatively, synthesis could occur indirectly via the release of another mediator.

We have now shown IL-1-like biological activity to be present in mini-pig epidermis. Anion exchange chromatographic purification of the epidermal samples led to recovery of the biological activity in the pI4–5 region, where porcine IL-1$\alpha$ would be expected to elute. One explanation for the absence of such activity in the neutral region could be that only trace amounts of IL-1$\beta$ are present in porcine epidermis. Measurement of small quantities of this cytokine would be further hindered by the lower potency of porcine IL-1$\beta$ relative to IL-$\alpha$ in the EL4-NOB-1 assay.[18] Alternatively, IL-1$\beta$ could be present in the unprocessed, precursor, form which is biologically inactive.[20] Others have reported the presence of IL-1$\alpha$ in porcine epidermis using immunohistochemical methods and a purified antiserum to porcine IL-1$\alpha$.[21] An antiserum to IL-1$\beta$ was not used. Negative staining was obtained in porcine stratum corneum in this study, which contrasts with our finding of IL-1-like biological activity.

Injurious stimuli, including ultraviolet light, have been reported to induce the release of IL-1 from human keratinocyte membranes *in vitro*.[8,9] Furthermore endothelial cells can generate PGs when incubated with supernatants from irradiated keratinocytes which contain IL-1.[22] If the PG formation that occurs in porcine skin after topical application of compounds such as B.Cl or HN is brought about by IL-1 mobilization from the epidermis and subsequent endothelial cell stimulation, a decrease in the epidermal IL-1 content might be expected. No significant difference was, however, observed between the amounts of IL-1 in vehicle treated sites, at which only slight, transient, increases in blood flow were detected in some pigs, and inflamed skin to which HN or B.Cl had been applied. One possible explanation may be that the amounts of IL-1 required to induce PG release are very small and hence any differences are obscured by the inter-site variation in IL-1 recoveries. Alternatively IL-1 could be released within the epidermal compartment to interact with cells activated by HN or B.Cl since, *in vitro*, IL-1 has been shown to induce PG release from human keratinocytes.[11] Further studies will be required to establish the nature of any interaction between IL-1 and PG formation *in vivo* in porcine skin during the inflammatory response to nicotinates and chemical irritants.

### References

1. Dowd PM, Whitefield M, Greaves MW. Hexyl-nicotinate induced vasodilation in normal human skin. *Dermatologica* 1987; **174:** 239–243.
2. English JSC, Winkelmann RK, Louback JB, *et al.* The cellular inflammatory response in nicotinate skin reactions. *Br J Dermatol* 1987; **116:** 341–349.
3. Barr RM, Brain SD, Camp RDR, *et al.* Levels of arachidonic acid and its metabolites in the skin in human allergic and irritant contact dermatitis. *Br J Dermatol* 1984; **111:** 23–28.
4. Plummer NA, Hensby CN, Kobza Black A, *et al.* Prostaglandin activity in sustained inflammation of human skin before and after aspirin. *Clin Sci Mol Med* 1977; **52:** 615–620.
5. Gahring LC, Buckley A, Daynes RA. Presence of epidermal-derived thymocyte activating factor/interleukin-1 in normal human stratum corneum. *J Clin Invest* 1985; **76:** 1585–1591.
6. Hauser C, Saurat J-H, Schmitt A, *et al.* Interleukin-1 is present in normal human epidermis. *J Immunol* 1986; **136:** 3317–3323.
7. Luger TA, Stadler BM, Luger BM, *et al.* Murine epidermal cell-derived thymocyte-activating factor resembles murine interleukin-1. *J Immunol* 1982; **128:** 1247–1252.
8. Sauder DN, Carter CS, Katz SI, *et al.* Epidermal cell production of thymocyte activating factor (ETAF). *J Invest Dermatol* 1982; **79:** 34–39.
9. Kupper TS, Chua AO, Flood P, *et al.* Interleukin-1 gene expression in cultured keratinocytes is augmented by ultraviolet irradiation. *J Clin Invest* 1987; **80:** 430–436.
10. Blanton RA, Kupper TS, McDougall JK, *et al.* Regulation of interleukin-1 and its receptor in human keratinocytes. *Proc Natl Acad Sci USA* 1989; **86:** 1273–1277.
11. Pentland AP, Mahoney MyG. Keratinocyte prostaglandin synthesis is enhanced by IL-1. *J Invest Dermatol* 1990; **94:** 43–46.
12. Albrightson CR, Baenziger NS, Needleman P. Exaggerated human vascular cell prostaglandin biosynthesis mediated by monocytes: Role of monokines and interleukin-1. *J Immunol* 1985; **135:** 1872–1877.
13. Lavker RM, Dong G, Sheng P, *et al.* Hairless micropig skin. A novel model for studies of cutaneous biology. *Am J Pathol* 1991; **138:** 687–697.
14. Chan CC, Ford-Hutchinson A. Effects of synthetic leukotrienes on local blood flow and vascular permeability in porcine skin. *J Invest Dermatol* 1985; **84:** 154–157.
15. Fourtanier A, Berrebi C. Miniature pig as an animal model to study photoaging. *Photochem Photobiol* 1989; **50:** 771–784.
16. Morris GM, Hopewell JW. Epidermal cell kinetics of the pig: a review. *Cell Tissue Kinet* 1990; **23:** 271–282.
17. Gearing AJH, Bird CR, Bristow A, *et al.* A simple sensitive bioassay for interleukin-1 which is unresponsive to $10^3$ U/ml of interleukin-2. *J Immunol Methods* 1987; **99:** 7–11.
18. Bird TA, Gearing AJH, Saklatvala J. Murine interleukin-1 receptor: differences in binding properties between fibroblastic and thymoma cells and evidence for a two-chain receptor model. *FEBS Letts* 1987; **225:** 21–26.
19. Fincham NJ, Camp RDR, Gearing AJH, *et al.* Neutrophil chemoattractant and IL-1-like activity in samples from psoriatic skin lesions. Further characterisation. *J Immunol* 1988; **140:** 4294–4299.
20. Mosely B, Urdal DL, Prickett KS, *et al.* The interleukin-1 receptor binds the human interleukin-1-$\alpha$ precursor but not the interleukin-1-$\beta$ precursor. *J Biol Chem* 1987; **262:** 2941–2944.
21. Ceriani CM, Murphy G, Kupper TS, *et al.* Detection of porcine interleukin-1-$\alpha$ in normal and corticosteroid-treated porcine skin. *J Invest Dermatol* 1989; **92:** 411.
22. Lim HW, Baker-Owens E, Sarmalkar M. UVB irradiation of keratinocytes induces generation of eicosanoids from endothelial cells. *J Invest Dermatol* 1990; **94:** 550.

ACKNOWLEDGEMENTS. This work was carried out with grant support from The Wellcome Trust (HDM). We would like to thank Mr. N Fincham and Professor RDR Camp for performing the anion exchange chromatography.

**Received 10 October 1991;
accepted in revised form 23 December 1991**
